# Pharmacokinetics of Two Chlorine-Substituted Bis-Pyridinium Mono-Aldoximes with Regenerating Effect on Butyrylcholinesterase

**DOI:** 10.3390/molecules25051250

**Published:** 2020-03-10

**Authors:** Huba Kalász, Zoltán Szimrók, Gellért Karvaly, Jennifer Adeghate, Kornélia Tekes

**Affiliations:** 1 Department of Pharmacology and Pharmacotherapy, Semmelweis University, Nagyvárad tér 4, 1089 Budapest, Hungary; szimrokz@gmail.com (Z.S.); jen.adeghate@gmail.com (J.A.); 2 Kalász Teaching and Research Co., Gvadányi utca 44-46, 1144 Budapest, Hungary; 3 Department of Laboratory Medicine, Semmelweis University, Nagyvárad tér 4, 1089 Budapest, Hungary; karvaly.gellert_balazs@med.semmelweis-univ.hu; 4 Department of Ophthalmology, University of Pittsburgh School of Medicine, Suite 820, Eye & Ear Building, 203 Lothrop Street, Pittsburgh, PA 15213, USA; 5 Department of Pharmacodynamics, Semmelweis University, Nagyvárad tér 4, 1089 Budapest, Hungary; drtekes@gmail.com

**Keywords:** butyrylcholinesterase, pyridinium aldoxime, RP-HPLC, distribution, K-203, K-867, K-870

## Abstract

Our aim was to find chlorine-substituted antidotes against organophosphate poisoning and compare their pharmacokinetics to their parent compound, K-203. White male Wistar rats were intramuscularly injected with K-203, K-867 or K-870. Serum, brain, kidneys, liver, lung, eyes, and testes tissues were taken after 5, 15, 30, 60, and 120 min and analyzed using reversed-phase high-performance liquid chromatography. K-203, K-867, or K-870 was present in every tissue that was analyzed, including the serum, the eyes, testes, liver, kidneys, lungs, and the brain. The serum levels of K-867 and K-870 (chlorine-substituted derivatives of K-203) were nearly constant between 15 and 30 min, while their parent compound (K-203) showed peak level at 15 min after the administration of 30 µmol/rat. Neither K-203, nor K-867 or K-870 were toxic at a dose of 100 µmol/200 g in rats. Chlorine-substitution of K-867 and K-870 produced limited absorbance and distribution compared to their parent compound, K203.

## 1. Introduction

Sarin and certain other phospho-organic compounds have been used by terrorists. The use of sarin [1,2,3] produced a horrifying outcome following a terrorist attack at a Tokyo metro station and in Syria. Not only the victims but also the rescue workers were in danger. Phospho-organic pesticides may also have serious side effects when they are applied improperly. The use of pralidoxime (a mono-pyridinium aldoxime) helps to restore acetylcholinesterase activity. Atropine, fluids, oxygen, and pralidoxime are used in the therapy [4].

The determination of pyridinium aldoximes-type cholinesterase reactivators in tissues can be done using either one of the detection such as UV absorbance [5,6,7,8,9], mass spectrometry [10,11], or an electrochemical detection [12,13], etc. By HPLC-mass spectrometry (HPLC-MS), Sakurada et al. [10] proved that a pyridinium aldoxime (pralidoxime) penetrates through the blood–brain barrier and constitutes a definite level in the brain. Our experimental work made it clear that the brain, the eyes, and the testes also contain a certain level of such xenobiotics after their application [14,15,16,17].

Kuca et al. [18,19,20,21,22,23] synthesized a large series of pyridinium aldoximes. A variety of compounds was produced, whose potency of reactivation was comparable to that of pralidoxime and obidoxime. Their pharmacokinetics were studied using rats. These compounds gave their maximum levels between 5 and 30 min, and showed a definite decline well before 60 min following intramuscular administration. This is the reason why rescue persons put an antidote in their regular package and use them on the site of intoxication. If several poisoned persons are being treated, the time of action of these pyridinium antidotes may not be enough for helping on the site. This is the reason why the pharmacokinetics of these compounds presents an essential tool when the best compounds of a large supply should be selected. A more or less retarded antidote has been scouted with the help of pharmacokinetics.

**Figure 1:**
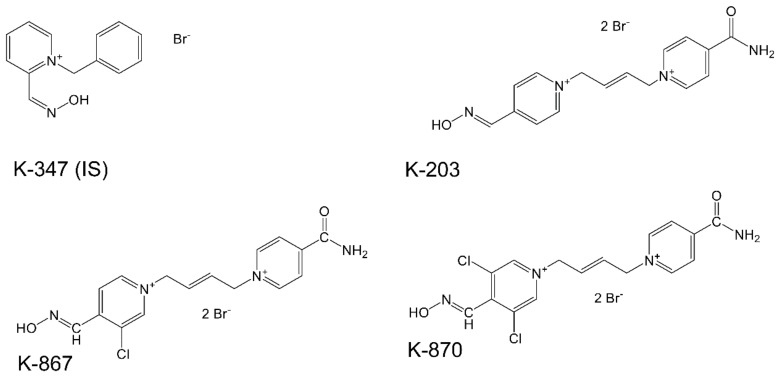
Chemical structures of K-347(IS), K-203, K-867 and K-870.

Pharmacokinetics of K-203 were studied in our previous works [8,14] as well as those of Karasova et al. [18]. Pyridinium aldoximes penetrate through blood-brain-barrier as it was detailed by Lorke et al. [24]. Both K-867 and K-870 are also pyridinium aldoximes, chlorine-substituted derivatives of K203, as shown in Figure 1.

Zorbaz et al. [25] determined the BChE reactivating concentrations of various chloride-substituted pyridinium aldoximes. Both K-867 and K-870 showed an adequate regenerating effect in a certain concentration on sarine-poisoned BuChE in vitro.

A search for reactivators with an adequately long level in the blood may be required. This paper demonstrates the success in finding chlorine-substituted compounds, simply by the use of an adequately increased dose.

## 2. Results

**Figure 2:**
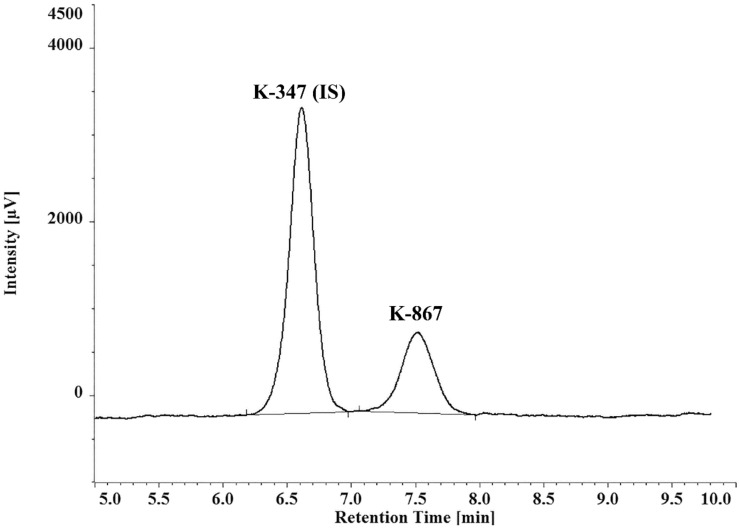
Chromatogram of a representative separation of K-347 (IS) and K-867.

Reversed-phase HPLC separation of either K-867 or K-870 was carried out using a relatively short C-18 column and ultraviolet detection. A demonstrative chromatogram of separation of K-347 and K-867 is given in Figure 2. Adequate separations of K-203, K-867, and K-870 were found from the matrix/background peaks of serum, and from the internal standard: K-347. Limit of quantitation (LOQ) values are: 1.09 µmol/L for K203; 1.01 µmol/L for K867; 0.95 µmol/L for K870.

**Figure 3:**
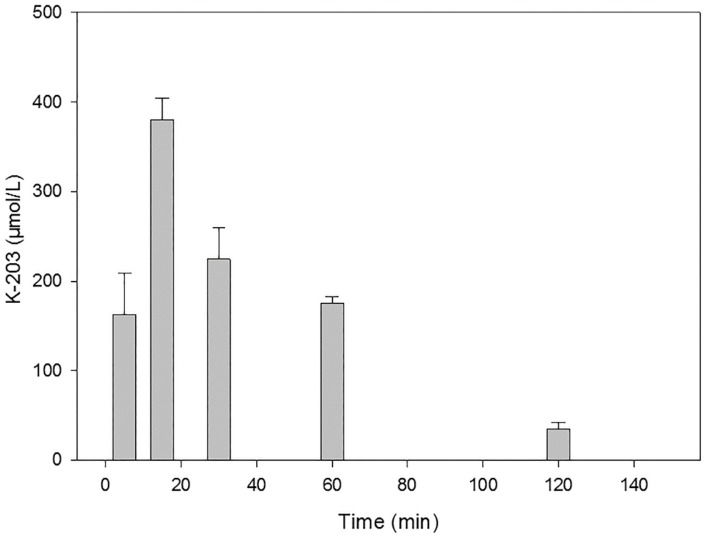
Time course of K-203 in serum following its intramuscular administration of 30 µmol.

**Figure 4:**
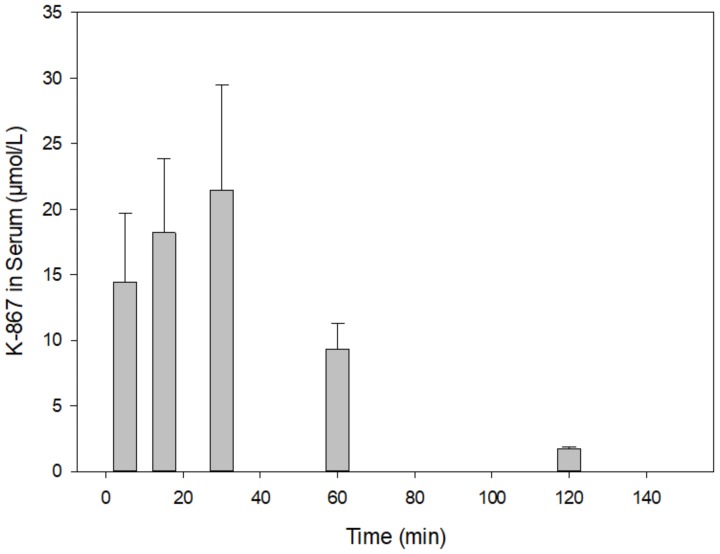
Time course of K-867 content in serum following its intramuscular administration of 3 µmol. The results show the means and standard deviations obtained in 4 parallel treatments. The displayed concentrations are the means of 5 HPLC determinations each.

**Figure 5:**
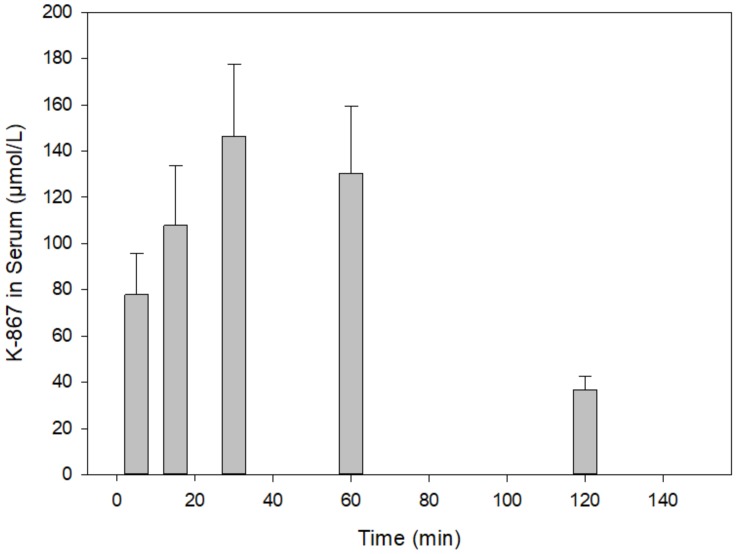
Time course of K-867 content in serum following its intramuscular administration of 30 µmol. The results show the means and standard deviations obtained in 4 parallel treatments. The displayed concentrations are the means of 5 HPLC determinations each.

**Figure 6:**
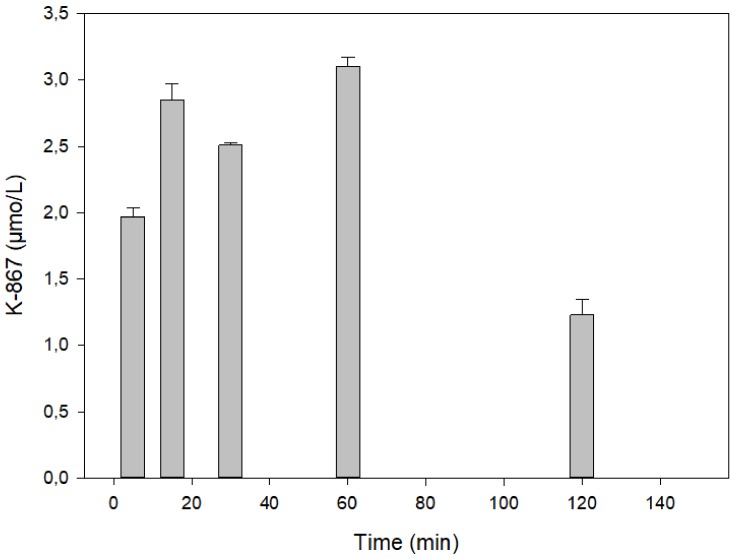
Time course of K867 content in the brain following its intramuscular administration of 30 µmol.

Figure 3 presents a time course in rat serum following the intramuscular injection of 30 µmol of K-203. The maximum of its level was reached at 15 min, which practically declined by its half at 30 min. A similar picture is shown in the case of K-867 when a dose of 3 µmol has been used (Figure 4). On the contrary, no decline in content was found before 60 min when 30 µmol K-867 was administered (Figure 5). This pharmacokinetic picture is also characteristic for the brain (Figure 6). The penetration of K-867 into the eyes and testes is higher (not shown here) than the level that penetrated into the brain.

**Figure 7:**
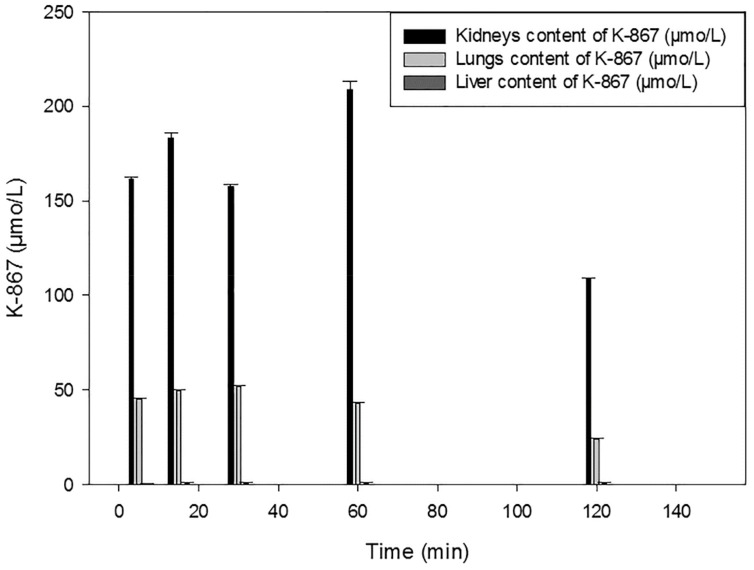
Time course of K-867 content in the kidneys, lungs and liver following its intramuscular administration of 30 µmol.

The elimination of K-867 mainly takes place through the kidneys; the lungs have a minor elimination rate, and the level eliminated through the liver is negligible. Figure 7 shows time versus K-867 concentrations in the kidneys, the liver and lungs following the administration of 30 µmol of i.m. injected K-867.

Administration of 3 µmol of K-870 resulted in low level in various tissues of the rats. This was the reason that 100 µmol the compound was injected i.m.

**Figure 8:**
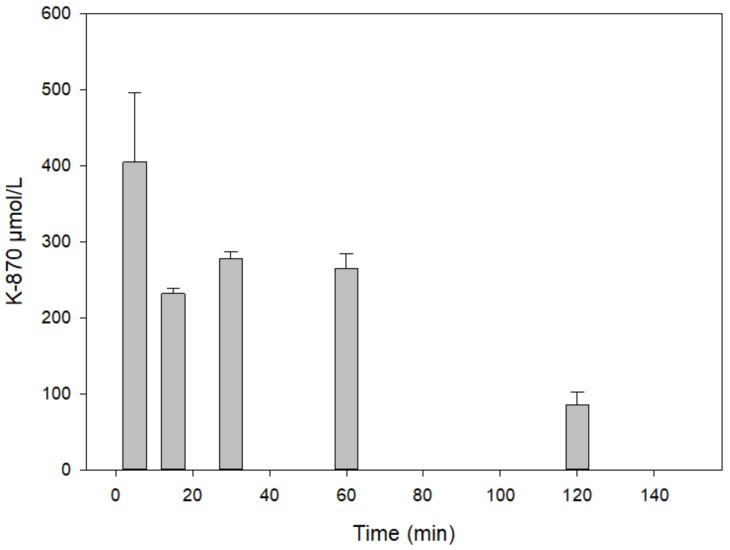
Time course of K870 content in the serum following its intramuscular administration of 100 µmol.

Figure 8 shows the serum level of a high dose (100 µmol) of K-870 calculated from the averages of two parallel in vivo experiments and three parallel chromatographic (RP-HPLC) determinations. Dose-dependence of K-870 mirrors the higher doses result in larger serum level.

Area under the curve (AUC) values were as follow:

K-203 (i.m. dose: 30 µmol) 21,086 µmol min L^−1^
K-867 (i.m. dose: 3 µmol) 1,287 µmol min L^−1^
K-867 (i.m. dose: 30 µmol) 12,199 µmol min L^−1^
K-870 (i.m. dose: 100 µmol) 27,227 µmol min L^−1^

## 3. Materials and Methods

K-203, K-347, K-867, and K-870 were produced in the Department of Chemistry at the Faculty of Sciences, University of Hradec Kralove, Hradec Kralove, Czech Republic. The chemical structures of K-347 (IS, internal standard), K-203, K-867, and K-870 are given in Figure 1.

Solvents and other chemicals: Acetonitrile (HPLC gradient grade from Merck, Darmstadt, Germany) and methanol (HPLC gradient grade from Merck, Darmstadt, Germany) were part of mobile phase for HPLC separations. The applied water was ultrapure, produced by a Millipore Ultrapure (Type 1) equipment, Merck Kft., Budapest, Hungary.

Animals: White male rats (180-199 g) were supplied by Toxicoop Co., Budapest, Hungary. They were intramuscularly injected with the freshly dissolved aqueous solution of either K-203, K-867 or K-870 using an adequate dose of the active compound. After 5, 15, 30, 60, and 120 min of administration, the anaesthetized animals were sacrificed. Animal treatment was done according to the ethical regulation of Semmelweis University and permitted by the Government Office of Pest County (No. PE/EA/385-7/2018).

Various tissues and body fluids were taken. The samples were immediately placed and kept on ice, then homogenized using 0.3 M of perchloric acid (PCA; ACS reagent grade, Sigma-Aldrich, St. Luois, MO, USA). A nine-fold excess of PCA was used to rat serum, while four-fold excesses were used for homogenization of all other tissue samples. Centrifugation was done with the homogenized samples and the supernatants were directly used for RP-HPLC as detailed earlier [26].

The employed HPLC system included a JASCO AS-4050 automatic injector, a JASCO PU-4180 pump with built-in degasser, and an MD-4010 photodiode array detector set at 300 nm and a JASCO ChromNav 2.0 software (ABL&E-JASCO Hungary Ltd., Budapest, Hungary). The analytes were baseline-separated on a Phenomenex Kinetex EVO-C18 100 × 3mm (5 µm) (Gen-Lab Kft. Budapest, Hungary) column (kept at 40 ℃) using reversed phase ion-pair chromatography in an isocratic run lasting 40 min. The mobile phase consisted of sodium acetate (5.44 g/L) (a.r., Molar Chemicals Kft., Budapest, Hungary), citric acid (4.72 g/L) (a.r., Reanal Laborvegyszer Kereskedelmi Kft., Budapest, Hungary) and 1-octanesulfonic acid sodium salt (2.0 g/L) (98%, Sigma Aldrich Kft., Budapest, Hungary).

K347 was employed as the internal standard (IS). IS was applied at the construction of the calibration curve, as well as to each sample before homogenization. Five (5) parallel injections were administered, 20 microliters each. The means of their results are used. Validation corresponds to the guideline of the European Medicine Agency (EMEA/CHMP/EWP/192217/2009 Rev. 1 Corr. 1**). European Medicine Agency, 2012. Available from: https://www.ema.europa.eu/documents/scientific-guideline/guideline-bioanalytical-method-validation_en.pdf. Accessed: 27 November 2019). Validation is detailed in our recent publication [27].

## 4. Conclusions

The fast elimination of antidotes is a general but not exclusive requirement. This is the case in a terrorist attack when the source of a toxic agent cannot always be eliminated. In general, the victims of a terrorist attack are immediately transferred to a safe place, however, rescue persons should take care of the victims while they are on the site of the attack. For the completion of their task, a long-lasting reactivator may be advantageous. Similarly to the pharmacokinetics of K-203 [8,14], chlorine-substituted bis-pyridinium aldoximes can also have a tissue level maximum at 15-min following intramuscular administration with a monotonous decline afterwards when 3 µmol is injected to rats of nearly 200 g. However, increasing the dose to 30 µmol, this decline is replaced to a near plateau (slight increase in serum concentration) through 5 to 30 min.

Substitution with chloride does not only reduce the solubility of K-867 and K-870, but also their absorption and distribution in circulation after intramuscular injection. This is evident from the fact that the AUC of the serum level of K-203 is 1.7 times more than that of K-867 when compared to the parent compound. In addition to the reduced absorption and distribution of K-867 in the body, its elimination is also impaired. An increase in the dose (from 3 to 30 micromole) of K-867 results in a protracted serum level of the compound.

The serum level of any xenobiotic compound influences the amount that gets into the central compartment of the body. It gives a possibility to the drug to penetrate into other compartments, such as body fluids, the central nervous system as well as the organs responsible for the elimination of the drug from the body. Both K203 and many other bis-pyridinium aldoximes (such as K-027, K-048, K-074, K-075, K-867, K-870) show definite penetration into the brain [16]. K-867 and K-870 open up new horizons for the production of antidotes. The shape of their serum and brain levels can be changed by increasing the dose.
